# Non-invasive and quantitative methods for assessment of blood flow in periodontal and oral soft tissues: a systematic review

**DOI:** 10.3389/fdmed.2025.1587821

**Published:** 2025-05-22

**Authors:** Amanda Rodriguez, Oliver Kripfgans, Fabiana Aellos, Diego Velasquez, Alejandra Baltazar, Hsun-Liang Chan

**Affiliations:** ^1^ Department of Periodontics and Oral Medicine, School of Dentistry, University of Michigan, Ann Arbor, MI, United States; ^2^ Department of Periodontics, College of Dentistry, University of Illinois Chicago, Chicago, IL, United States; ^3^ Department of Radiology, Michigan Medicine, University of Michigan, Ann Arbor, MI, United States; ^4^ Department of Surgery, Stanford School of Medicine, Stanford University, Stanford, CA, United States; ^5^ Private Practice, Fenton, MI, United States; ^6^ Division of Periodontology, College of Dentistry, The Ohio State University, Columbus, OH, United States

**Keywords:** ultrasound, tissue perfusion, microvasculature, laser speckle, laser Doppler flowmetry, non-invasive

## Abstract

**Objectives:**

Understanding the available methods to study blood flow in the oral cavity can enhance knowledge of research methodology on periodontal circulation related to disease initiation and progression as well as wound healing. This study aims to systematically review non-invasive techniques that allow for the assessment of oral tissue perfusion in clinical and pre-clinical studies.

**Methods:**

A complete electronic literature search in 5 databases (NLM PubMed, Embase, EBSCOhost CINAHL, EBSCOhost Dentistry and Oral Sciences Source, and Wiley Cochrane Central Register of Controlled Trials) was conducted by two reviewers. The search terms included gingival blood flow, tissue perfusion, imaging perfusion, soft tissue perfusion, diagnostic, vascularization, soft tissue, and microvascularization. The focused question is: What are the available non-invasive and quantitative imaging techniques used to evaluate oral and periodontal tissue perfusion?

**Results:**

A total of 79 articles were included for qualitative analysis. Various methods were identified, including Laser Doppler Flowmetry (LDF), Laser Speckle Contrast Imaging (LSCI), Spectral Imaging Methods (such as Diffuse Reflectance Spectroscopy), Ultrasound (US), Intravital Video Microscopy, and Oral Videocapillaroscopy. LDF is the most applied to estimate blood flow in a small focal area for the study of periodontal diseases and oral wound healing, among other indications. LSCI, providing surrogate superficial blood flow values in a 2-dimensional, larger field-of-view, has been used for similar reasons. The use of cross-sectional ultrasound is on a rise to record blood velocity and blood volume using color flow and color power modes, respectively. Comparisons of the available technologies revealed their strengths and limitations related to their spatial resolution, sensitivity, reliability, accuracy, invasiveness, dependence of (image) data in the field of view relative to probe positioning and angulation, and safety. The ideal features of such a device pertinent to probe geometry, data acquisition, recording, and infection control needs were also discussed.

**Conclusions:**

A few imaging technologies have been identified in the literature to study blood flow in the oral cavity. These methods could potentially augment our ability to diagnose oral diseases and monitor wound healing objectively and timely. In combination, these could potentially enhance treatment outcomes significantly.

## 1 Introduction

Anatomical variability of oral and periodontal microcirculation and its reaction to the outside stimuli, such as bacterial pathogens and surgical trauma have been a topic of interest for the diagnosis of oral diseases and outcome evaluation of dental treatment. Anatomical studies in human cadavers and animals have provided a profound insight into oral soft tissue perfusion (1–5). Recent studies have reiterated the apico-coronal orientation of the microvascular plexus and its intricately interconnected network of vessels as the key supplier to the mucogingival complex (1, 6, 7). More specifically, these principal micro-vessels, the supraperiosteal, intra-ligamental, and intra-osseous plexuses, have their own territories yet extensively interconnected. However, the spatial distribution of the supraperiosteal plexus is not well-defined. A recent study suggested the distribution is random and varies individually (7). As far as surgical wound healing is concerned, vascular distribution and inclusion in the flap, and the degree to which microvasculature is traumatized can influence the speed of tissue re-perfusion postoperatively, wound stability, and ultimately outcome predictability (7–10). This knowledge could further assist the clinician in identifying strategies to preserve vascularization and consequently achieve optimal wound healing (7, 11).

In addition to anatomical evaluation of microvasculature, vascular circulation, such as the amount and speed of the blood flow can also provide valuable insight into oral disease diagnosis and tissue healing capabilities, potentially influencing clinical outcomes. Under normal healing events of a periodontal access flap, there is a short period of significant ischemia following local anesthesia and immediately post-operatively (12). A hyperemic response follows and persists for a few days (13). In general, the flow returns to the baseline between the 1st and 2nd week. Deviation from this normal pattern may suggest altered healing that warrants attention. For example, delayed reperfusion could indicate compromised and inferior healing. Prolonged tissue hyper-perfusion may be related to the occurrence of infection and sustained inflammation. This dynamic shift in the amount of periodontal tissue perfusion may also be influenced by incision tracing and flap designs (13, 14). The simplified papilla preservation flap may be associated with faster recovery of the gingival blood flow post-operatively compared to the modified Widman flap (15). Other factors, such as a minimally invasive approach, could also affect tissue perfusion and wound healing. Microsurgery for treating gingival recession with the use of the operating microscope has been shown to encourage faster revascularization and better root coverage outcomes (16). Clinical methods for quantitative evaluation of the baseline as well as postoperative tissue perfusion have not been established.

Several techniques have been used to estimate and study tissue perfusion in the past few decades (1, 2, 6, 9–12, 15, 17–34). Laser Doppler Flowmetry (LDF) for example, can provide relative blood flow at a focal area of a few millimeters in diameter. Laser Speckle Contrast Imaging (LSCI) also offers relative superficial blood flow and image display showing the perfusion intensity on a scale, with the region of interest covering a few teeth. Recently, the use of ultrasound to estimate tissue perfusion has been increasing. The two main flow imaging modes are color flow and power mode, quantifying flow velocity and blood volume, respectively. The literature suggests that monitoring tissue perfusion at the individual level could prove to be more valuable instead of differentiating the mean perfusion amount of the cohorts (18, 28). Various techniques offer valuable information in different aspects, depending on their uses and indications. Realizing and contrasting these available modalities could lay the foundation for studying oral and periodontal tissue perfusion, disease pathogenesis and progression, as well as wound healing because of different surgical concepts, designs, or conditions. Therefore, the present study aims to systematically review non-invasive imaging techniques that quantitatively assess oral and tissue perfusion.

## 2 Materials and methods

The Preferred Reporting Items for Systematic Reviews and Meta-Analyses (PRISMA) statement was consulted for the present systematic review process, which complies with the appropriate guidelines/checklist (35) (Supplementary File).

Focused question:

What are the available non-invasive and quantitative imaging techniques used to evaluate oral and periodontal tissue perfusion?

### 2.1 Eligibility criteria

#### 2.1.1 Inclusion criteria

• Human as well as pre-clinical *in vivo* studies that used imaging technology to quantify oral and periodontal tissue circulation.

#### 2.1.2 Exclusion criteria

• *Ex vivo* studies.
• Review articles, including workshop consensuses.
• Retrospective studies.
• Articles not written in English.

### 2.2 Search strategy

An electronic search was conducted by two reviewers (A.R. and F.A.) from January 2022 to October 2023 in the following databases: NLM PubMed, Embase, EBSCOhost CINAHL, EBSCOhost Dentistry and Oral Sciences Source, and Wiley Cochrane Central Register of Controlled Trials. The working PubMed search was as follows: (((gingival blood flow) AND (tissue perfusion)) OR (imaging perfusion)) OR (soft tissue perfusion) AND ((diagnostic) OR (vascularization)) OR (soft tissue)) OR (microvascularization))). A second PubMed search was performed to include the following search terms: ((soft tissue) OR (tissue)) AND ((blood flow) OR (perfusion) OR (vascularization)) AND (diagnostic) AND (imaging) AND (oral) AND (gingival).

### 2.3 Data extraction and analysis

Data were manually extracted by the same examiners (A.R. and F.A.) independently. When they disagreed, a third (D.V.), fourth (H.L.C.), fifth (A.B.), and sixth examiner (O.D.K.) checked the variable and a consensus agreement was reached. The Preferred Reporting Items for Systematic Reviews and Meta-Analyses (PRISMA) guidelines/checklist were followed during the present systematic review. Two researchers (A.R., F.A.) independently assessed the quality of the included studies. In cases of disagreement, a third, fourth, fifth, and sixth researcher (D.V., H.L.C., A.B., O.D.K.) were consulted. Due to the heterogeneity of the evaluated parameters, time points, and clinical or preclinical scenarios, a meta-analysis could not be performed.

### 2.4 Risk of bias assessment

The quality analysis process was based on the updated version of the *Handbook for Systematic Reviews of Interventions* and the CONSORT (Consolidated Standards of Reporting Trials) statement (36). Study characteristics, quality, and the risk-of-bias tool (ROBINS-I) for non-randomized studies of interventions (non-RCTs) (37), and RoB2 for randomized clinical trials (RCT) (38). These assessments were conducted independently by two authors (A.R.B. and F.A.), with final evaluations reached through discussion. The degree of bias was categorized as follows: (1) low risk if one criterion was missing; (2) moderate risk if two or three criteria were missing; (3) serious risk if three or more criteria were missing, and (4) critical if four or more criteria were missing.

## 3 Results

### 3.1 Study selection

**Figure 1:**
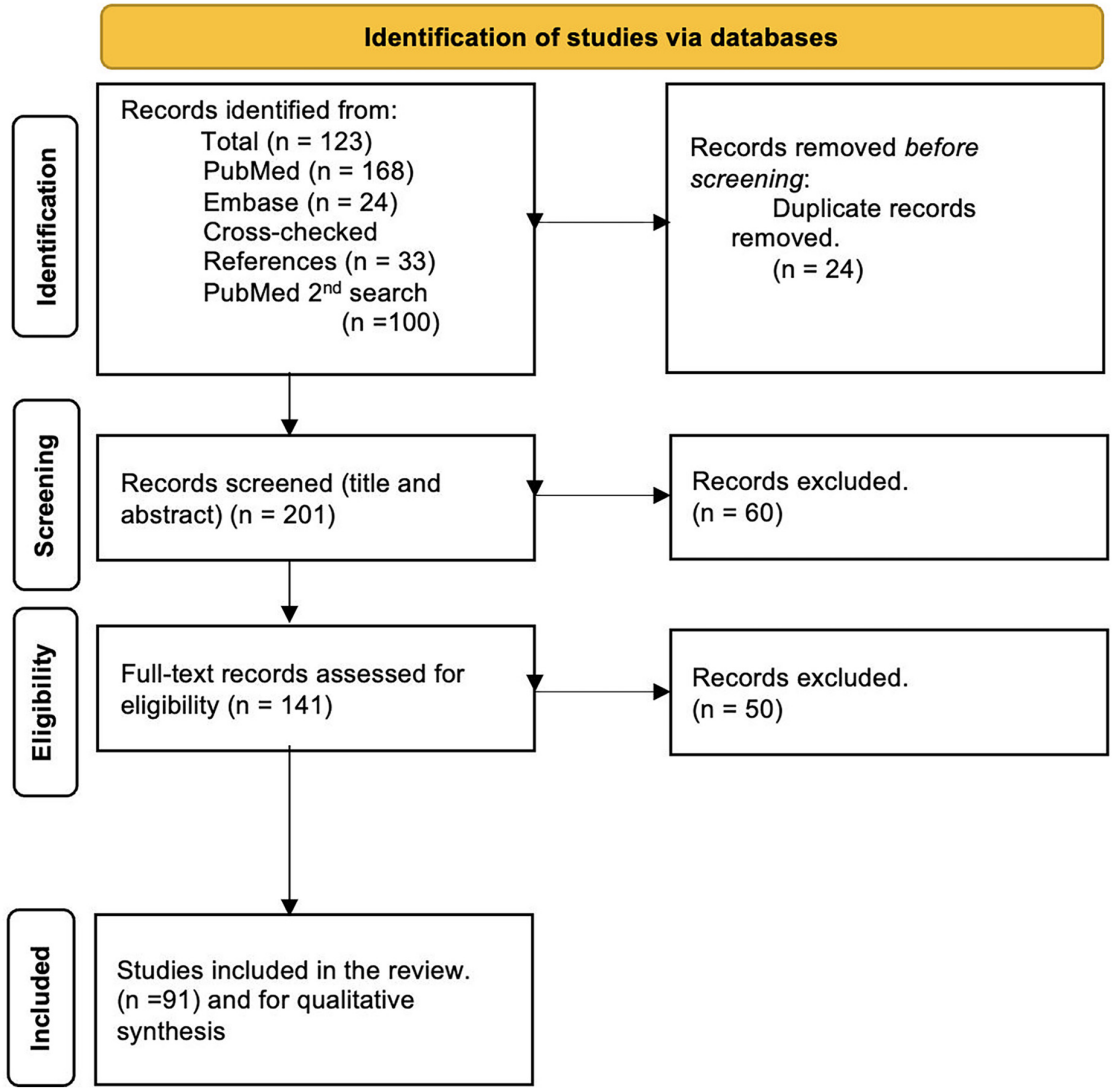
PRISMA flowchart illustrating the literature selection process.

The literature selection process is illustrated by a PRISMA flowchart (Figure 1). Initial screening yielded a total of 223 articles, 68 in PubMed, 24 in the EMBASE database search, 33 cross-checking references, and a second PubMed search yielded 100 articles. References from these searches were combined, and after removing 24 duplicates, 201 articles were available for title and abstract review. Of these articles, 60 did not meet the inclusion criteria and were excluded. Following a full-text review of the remaining 141 articles, 50 articles were further excluded, resulting in 91 included articles. An overview of the identified techniques is given in Table 1. For each imaging mechanism, a summary table is provided with technique-specific details: Laser Doppler Flowmetry (LDF) (Table 2), Laser Speckle Contrast Imaging (LSCI) (Table 3), Spectral Imaging Methods (Table 4), Ultrasound (US) (Table 5), and other imaging methods (Videocapillaroscopy, Videomicroscopy, Optical coherence tomography (OCT) with OCT angiography (OCTA), real-time optical vascular imaging (RTOVI), Narrowband imaging (NBI) (Table 6). Considerable heterogeneity was found in the evaluated parameters, which precluded quantitative data analysis.

**Table 1:** Overview of *in vivo* oral perfusion techniques.

Imaging techniques	Mechanism	Signal source	Field of view/ROI	Penetration Depth	Primary outputs		
					RBC velocity	Capillary characters, such as morphology density/diameter	Composition/property, such as Oxygenation, Hb concentration
Optical technology							
Laser Doppler Flowmetry (LDF)	Detecting the Doppler shift of laser light scattered by Hemoglobin (Hb) in moving red blood cells (RBC)	Diode laser with light wavelength of 630, 780 or 830 nm	Generally, 1 mm diameter	Up to 1 mm	X	Laser Doppler Flowmetry (LDF)	Detecting the Doppler shift of laser light scattered by Hemoglobin (Hb) in moving red blood cells (RBC)
Laser Speckle Contrast Imaging (LSCI)	Measure speckles that are produced when coherent light is scattered back from Hb of RBCs	Diode laser with infrared light	Depends on zoom power, in general 10–40 mm	Up to 1 mm	X	Laser Speckle Contrast Imaging (LSCI)	Measure speckles that are produced when coherent light is scattered back from Hb of RBCs
Laser Doppler Spectrophotometry	Detecting the Doppler shift of laser light scattered by moving RBCs	Diode laser with wavelength of 630, 780, or 830 nm and white light (500–800 nm)	Generally, 1 mm diameter	Up to 1 mm	X	Laser Doppler Spectrophotometry	Detecting the Doppler shift of laser light scattered by moving RBCs
Diffuse Reflectance Spectral Imaging (DRS)	Measure reflection of light to analyze tissue composition	UV and visible light (180–800 nm)	8 × 8 mm	Up to 1 mm		Diffuse Reflectance Spectral Imaging (DRS)	Measure reflection of light to analyze tissue composition
Near-infrared spectroscopy (NIRS)	Measure near-infrared light reflection to measure tissue composition	NIR (780–2,500 nm)	Generally, a few mm	Few cm		Near-infrared spectroscopy (NIRS)	Measure near-infrared light reflection to measure tissue composition
Sidestream Dark Field (SDF)	Measure LED absorption/reflection by Hemoglobin in capillary	LED (530 nm)	Depends on zoom power, in general 1 mm	500 μm	X	Sidestream Dark Field (SDF)	Measure LED absorption/reflection by Hemoglobin in capillary
Orthogonal Spectral Polarization (OPS)	Measure green light absorption by Hemoglobin in capillary	Linearly polarized light (530 nm)	Depends on zoom power, in general 1 mm	Up to 1 mm	X	Orthogonal Spectral Polarization (OPS)	Measure green light absorption by Hemoglobin in capillary
Intravital Videomicroscopy (IVM)	Image cells illuminated by light under microscope	Visible spectrum plus ultra-violet and near-infrared wavelengths (∼300–1,100 nm)	Depends on zoom power, in general 1–3 mm	Up to 1 mm	X	Intravital Videomicroscopy (IVM)	Image cells illuminated by light under microscope
Video capillaroscopy (VC)	Image capillary illuminated by light under microscope	Visible spectrum (400–700 nm)	Depends on zoom power, in general 1–3 mm	Up to 1mm	X	Video capillaroscopy (VC)	Image capillary illuminated by light under microscope
Optical coherence tomography (OCT) and optical coherence angiography (OCTA)	Image blood vessels patterns and density by light waves	1,310 nm wavelength and 110 nm bandwidth	5.25 mm × 5.25 mm	Up to 2 mmm	X	Optical coherence tomography (OCT) and optical coherence angiography (OCTA)	
Real time optical vascular imaging technique (RTOVI)	Image microvascular features by green light	Wavelength (550 ± 10 nm)	0.51 × 0.51 mm	Up to 1 mm	X	Real time optical vascular imaging technique (RTOVI)	
Narrowband imaging (NBI)	Image mucosal surfaces and microvasculature by optical filters with light	Wavelenght filters vary 500, 445, and 415 nm with ∼30 nm bandwidth	Depends on zoom power, in general up to 100X	170–240 μm		X	
Imaging techniques							
Ultrasound	Measure sound frequency changes due to RBC movement	Sound of 12–25 MHz	Depends on probe size, in general 15–20 mm	Up to 15 mm	RBC velocity		

**Table 2:** Wound healing assessment by Laser Doppler flowmetry.

Author (year)	Study design	Aim	Indication	Time evaluated	Results	Clinical relevance
Heitzer et al., 2025	Pre-clinical	Compare the hemostyptic properties and gingival healing after tooth extraction in rodent under rivaroxaban therapy over 10 days	Hemostyptic properties and gingival healing between novel polyurethane based adhesive VIVO and gelatin sponge (GESP)	1 time point	Increase mean blood flow for both treatments	VIVO demonstrated effective hemostasis and favorable gingival healing following tooth extraction under continuous rivaroxaban therapy
Katz et al., 2024	Clinical	Evaluate peri-implant tissue perfusion (blood flow) in implants placed in pristine bone, avascular, and microvascular grafts	Gingiva; perfusion in different bone types in dental implants	1 time point	No differences found in gingival blood flow in either pristine or avascular bone or microvascular grafts	Perfusion was similar between implants in native and augmented sites, though those in avascular or microvascular grafts showed increased peri-implant inflammation
Diehl et al., 2022	Clinical	Assess microcirculation and the expression patterns of wound-healing related implants in type 2 diabetes mellitus patients	Periodontitis	3 months	Wound healing after implant surgery was similar in healthy patients and T2DM	Considered hydrophilic surface titanium zirconium with reduced diameter for T2DM patients
Liu et al., 2022	Pre-clinical	Measure changes in blood flow rate using advanced PRF in early-stage gingival regeneration after tooth extraction	Gingival tissue regeneration	1 time point	Gingival blood flow was higher with A-PRF	A-PRF may be beneficial for gingival tissue regeneration
Miron et al., 2022	Clinical	Evaluate microcirculation at marginal gingiva after change of toothbrush	Periodontal health	14 days	New toothbrushes in adolescents with healthy gingiva can increase gingival blood flow	Changing the toothbrush in less than a month could be a factor in gingival micro irritation
Svetlana et al., 2022	Clinical	Determine and compare dental pulp and gingival blood flow	Radiotherapy	6 months	RT cause a significant acute gingival blood flow	Protect teeth before radiotherapy to avoid ischemia of soft tissues
Komaki et al., 2022	Clinical	Investigate the hemodynamics of gingival microcirculation	Occlusal trauma	During and after clenching	Ischemia was present during clenching followed by reactive hyperemia by the release of clenching	Occlusal trauma should be prevented to avoid detrimental effects on periodontal tissues due to compression of the vascular network of the periodontal membrane
Laredo-Naranjo et al., 2021	Clinical	Identify gingival microcirculation	Orthodontic treatment	30 days	Longitudinal evaluation showed increased perfusion in Nitinol arches three times greater than basal flow	Identification of inflammatory process in treatment is crucial to discontinue the use of harmful methods
Yamamoto et al., 2021	Pre-clinical	Assess changes in peri-implant vascular network and blood flow	Peri-implant tissue inflammation	90 days	Significant increase in blood flow around implants with an inflamed blood vessels invade the bone marrow through the bone margin of the alveolar bone	Analysis of gingival microcirculation of tissues may aid the pathological analysis in clinical setting
Kuraji et al., 2019	Pre-clinical	Evaluate temporal changes in gingival blood flow during disease progression	Periodontitis	28 days	Levels of alveolar bone loss, gene expression and immunostained VEGF- positive vessels correlated with increase changes in gingival blood flow.	Dynamic alteration of gingival blood flow was demonstrated during disease progression, inflammation, vasculogenesis and alveolar bone resorption
Alssum et al., 2017	Clinical	Assess gingival blood perfusion and wound fluid	Post extraction regenerative	120 days	Transient increases in angiogenic and prolonged hyperemia of soft tissue	Soft tissue ischemia-reperfusion model does not determine radiographic bone changes
Kaner et al., 2017	Clinical	Characterize early phase of wound healing after periodontal surgery with and without Enamel matrix derivative (EMD)	Early wound healing after periodontal surgery	2 weeks before surgery and 14 days after surgery	Significantly decrease at the papillary base with increased blood flow until day 14. Group without EDM showed decreased blood flow at day 1 when compared	Detection of changes in microcirculation after surgery with differentiation between surgical techniques
Ogino et al., 2017	Clinical	Examined the extent of reactive hyperemia effect on GBF in denture-supporting mucosa during chewing	Biting force	After simulated biting or chewing for 30 s at 8 time points	Significant correlation between GBF chewing and the extent of reactive hyperemia	Slow chewing induced less GBF than regular or fast chewing in denture-supporting mucosa. Subject with less reactive hyperemia had less GBF in denture-supporting mucosa
Tatarakis et al., 2017	Clinical	Assess early healing process using a xenogenic collagen matrix (CMX) or connective tissue graft (CTG)	Root recession coverage	30 days	CTG showed a vascular homogeneous pattern whereas CMX had a second phase of increased blood flow at 14 days	Identify early vascular response and assessing maturity of surgical wound
Le Bars et al., 2016	Clinical	Measure the microcirculation of the healthy palatal mucosa at three specific points, and to test the reproducibility and sensitivity of the LDF	Palatal mucosa (median raphe (MR), Schroeder area (SA), retro incisive papilla (RP)	3 min	Palatal blood flow differed significantly from other surfaces. SA showed the highest GBF values followed by RP and MR	LDF can help to detect the onset of pathological alterations of the palatal mucosa
Reuther et al., 2016	Clinical	Investigate the effect of electronic cigarettes (with nicotine and without) on blood flow in the buccal mucosa	Electronic cigarette smoking	After 5 min of vaping. Intervals at 5 min for 30 min	Wide variation in results and a small but significant rise in nicotine vaping but fell to baseline within 30 min	Electronic cigarettes may influence blood flow in the oral mucosa
Svalestad et al., 2014	Clinical	Evaluate the effect of hyperbaric oxygen therapy (HBOT) on vascular function	Gingival mucosa	6 months	Vascular capacity increased by HBOT, and effect persists up to 6 months	A significant increase in maximal blood flow may indicate a vascular bed with improved healing capacity
Kawaai et al., 2013	Clinical	Assess the oral mucosal blood flow during sedation with dexmedetomidine	Palatal	0, 5, 10, 12, 22 and 32 min after the start of the infusion	GBF decreased significantly after the start of the infusion of dexmedetomidine	LDF showed decreased levels of GBF by the mediating effect of dexmedetomidine on a-2 adrenoceptors
Kozlov and Ibragim 2011	Clinical	Examine GBF in patients with varying degrees of inflammation in health, gingivitis and periodontitis	Periodontal disease	1 time point	Changes in micro vessels lead to the development of blood flow stagnation in post capillaries and local stasis in the gingival tissues	LDF can estimate the severity of the disorders of the microcirculation in the gingiva in the development of periodontitis
Okada et al., 2010	Clinical	Determine the effect of the number of biting forces on change in blood flow in denture-supporting maxillary mucosa.	Biting force	BL, at rest (1, 4, 8, 12 min)	GBF showed statistically significant differences at pre-loading and loading	There was an increase in mean blood flow during intermittent loading relative to at pre-loading
Sakr et al., 2010	Clinical	Characterize the buccal microvascular response in patients with septic shock	Septic shock	Every 2 s for 5 min and after 1 week	GBF increased during the 2nd day of septic shock and decreased after. Non septic patients GBF was significantly greater superficially	LDF may be useful for tracing microvascular alterations in critically ill patients
Svalestad et al., 2010	Clinical	Evaluate reproducibility of LDF for assessing microvascular blood flow after radiotherapy and hyperbaric oxygen therapy	Mandibular mucosa	6 weeks	Blood flow increased when compared to basal flow after heat provocation	LDF showed reproducibility in longitudinal studies
Singh et al., 2008	Clinical	Comparison between blood flow in the tongue and oral mucosa with 2 LDF probes	Intraoral cavity	3 time points (0, 6 and 24 h)	Measurements by the 2 probes were correlated significantly but the standard deviations were large	LDF measurements showed large variations between probes
Retzpei et al., 2007a	Clinical	Compared gingival blood flow responses followed simplified papilla preservation (test) and modified Widman flap (control)	Periodontal access flap	60 days	Results present an ischemia-reperfusion flap model with papilla preservation flap with faster recovery. A peak hyperemic response resolved by day 4 (test) but persisted until day 7 (control)	LDF may present clinical applicability in recording dynamic changes in the microcirculatory blood perfusion. Papilla preservation flap may be associated with faster recovery of gingival blood flow post-operatively
Retzepi et. 2007b	Clinical	Investigate pattern of gingival blood flow changes after periodontal access flap surgery	Periodontal access flap surgery	60 days	Different areas of the flaps consistently showed an ischemic-hyperemia patterns of perfusion alteration during wound healing	This technique may be useful in representing the dynamic nature of flaps blood flow reperfusion
Rodriguez-Martinez et al., 2006	Clinical	Explore possible association between an index of gingival microvascular perfusion response to compression of alveolar mucosa	Periodontitis	2 measurements 4 min apart	Microvascular density, dilation, elongation and organize capillary network has a positive correlation with clinical measurement and increased reactive hyperemia	Increase or decrease in gingival perfusion could indicate clinical predominance of gingivitis or periodontitis
Donos et al., 2005	Clinical	Evaluate the applicability of the LDF in recording changes in gingival blood flow	Periodontal surgery	60 days	GBF increases in comparison to baseline values until the 7th day. By the 15th day, 30th, and 60th gingival blood flow values were similar to the baseline	LDF might present clinical applicability in recording changes in gingival blood flow following periodontal surgery
Kocabalkan and Turgut 2005	Clinical	Investigate the influence resin base materials on the blood flow of underlying mucosa	Biting forces	Baseline (BL), 1 week, 1, 3 and 6 months	Soft lining and hard acrylic: Mean GBF in mucosa after 1 week was significantly lower than baseline and at 6 months after return to normality Hard: GBF in molar region increased after 6 months compared to BL	Dentures hinders blood flow to supporting tissues
Patino-Marin et al., 2004	Clinical	Systematize a procedure that allows characterization of perfusion response pattern to topical and transitory compression	Attached gingiva	2 measurements 5 min apart	Gingival compression propitiated an induced flow debt followed by increased flow after compression is released	Microvascular responses are reproducible indices of perfusion response whose validation under pathological circumstances remains to be evaluated
Kerdvongbundit et al., 2003	Clinical	Evaluate dynamic changes in the micromorphology and microcirculation of healthy and inflamed human gingiva	Gingival surfaces (free and attached gingiva, interdental papilla and alveolar mucosa)	15 min with 90 s intervals	GBF in healthy and gingivitis was significantly different. After treatment at 1 and 3 months GBF showed significant differences	LDF can be used to record GBF before and after inflammation reduction
Kemppainen et al., 2003	Clinical	Study capsaicin-evoked blood flow responses in maxillary gingiva	Alveolar mucosa and attached gingiva	4–15 s	Significant higher blood flow during the stimulation period and 3 min after	Alveolar mucosa is more sensitive to chemical irritants than attached gingiva
Akazawa and Sakurai 2002	Clinical	Investigate the influence of the continuous compression because of light clenching on the GBF of the denture underlying mucosa in tissue-supported or tooth-tissue-supported dentures	Biting force	Baseline and 5, 10, 20, 30 and 60 s	GBF in mucosa underlying the denture showed statistically significant correlation between recovery and loading time	Continuous clenching results in ischemia and delays the recovery of GBF in the mucosa after the release of compression
Ambrosini et al., 2002	Clinical	Evaluation of the modifications occurring in human gingival blood flow following periosteal stimulation	Surface of the gingival graft	Intervals of 1 week	Increased blood flow was shown at 7 days after periosteal stimulation	Detects alterations of the vascularization several hours before the clinical symptoms become apparent during wound healing monitoring
Vag and Fazekas 2002	Clinical	Monitor reactions of marginal gingiva during prosthetic rehabilitation	Prosthetic rehabilitation	6 weeks	Significant correlation was found between gingival index and increased blood flow	Monitoring gingival blood flow may provide valuable information of the healing process of inflamed marginal gingiva
Kerdvongbundit and Vongsavan et al., 2002	Clinical	Evaluate the microcirculation in the gingiva of healthy and gingivitis patients	Gingival surfaces (free gingiva, interdental gingiva, attached gingiva, and alveolar mucosa)	90 s intervals for 15 min	Blood flow in the maxillary anterior gingiva showed significant differences from the mandibular anterior gingiva in the interdental gingiva, attached gingiva, and alveolar mucosa	GBF in the maxillary anterior gingiva was greater than that in the mandibular anterior gingiva. GBF was lower in free gingiva and higher in alveolar mucosa
Kerdvongbundit and Vongsavan et al., 2002	Clinical	Evaluate the microcirculation in patients with moderate gingivitis, periodontitis, and healthy gingiva	Gingival surfaces (free and attached gingiva, interdental papilla and alveolar mucosa)	90 s intervals for at least 15 min	GBF in moderate gingivitis and periodontitis showed similar values and were higher than those in healthy gingiva	GBF in patients with periodontitis and gingivitis revealed significant differences when compared to healthy patients
Heckmann et al., 2001	Clinical	Study oral mucosal blood flow in burning mouth syndrome (BMS) patients	Hard palate, the tip of the tongue, midline of the oral vestibule, and lip	2 min	BMS patients exhibited a higher significantly response on the hard palate compared to healthy patients	Higher vascular reactivity in patients with burning mouth syndrome when compared to healthy patients
Matsuki et al., 2001	Clinical	Measure gingival blood flow under different water temperatures and evaluate reproducibility	Marginal gingiva	5 days	Warm water (36 and 50°C showed a significant increase in blood flow and flow went to baseline (after 3 and 4 min, respectively). Cold water (4°C) showed decreased blood flow and level went to baseline after 2 min.	LDF showed acceptable reproducibility with no significant effect from the probe angle
Heckmann et al., 2000	Clinical	Study the changes of blood flow after the effects of painful stimulation using dry ice (CO2)	Hard palate, lip and oral vestibule	Baseline and 2 min	Mucosal blood flow increased significantly at all sited after dry ice application	Mucosal blood flow varies at different mucosal sites. Tongue blood flow changes were the least pronounced
Ahn and Pogrel 1998	Clinical	Determine if 2% lidocaine with 1:100,000 epinephrine decreases the blood flow in gingival tissues	Gingival margin	Recording during 15 min and 1 min at 10 intervals	Significant decrease of GBF (maximum decrease 51%) with gradual return to baseline up to 35 min.	Gingival effects may be of relevance with soft tissue surgical procedures
Perry et al., 1997	Clinical	Quantify changes in blood flow following tooth brushing	Tooth brushing	4 weeks	Tooth bruising for 3 and 10 s significantly increased blood flow	LDF showed to be a non-invasive tool to understand dynamics of blood flow
Ketabi and Hirsch 1997	Clinical	Determine vascular responses in the gingiva of smokers and non-smokers after anesthetic injection	Local anesthetic	15 min	Significant decrease of GBF (average 46%) in the gingiva. Statistically significance for GBF recovery between smokers and non-smokers	LDF showed that recovery of GBF is longer in smokers than in non-smokers.
Schmid-Schönbein et al., 1997	Clinical	Identify vascular temporal patterns in mucosal microcirculation in maxillary gingiva with laser Doppler anemometry (LDA)	Gingivitis	12.5 min	Dynamic fluctuation relates to physiological events synchronized to neurodynamic activity or ventilatory influences, gravitational effects or arterial o venous pressure	Analysis of data obtained in the study allows temporal pattern characteristics for future diagnostic procedures
Herlofson et al., 1996	Clinical	Test the oral mucosa irritant potential of toothpaste detergent (sodium lauryl sulfate)	Gingiva	10–15 min	GBF increased significantly from the second to ninth minute	LDF may be a useful non-invasive technique for intraoral testing of different agents of intraoral use
Hinrichs et al., 1995	Clinical	(1) Determine gingival blood flow in the gingival sulcus after probing and injection of local anesthetic. (2) Determine reproducibility of measurements	Periodontal probing and after local anesthetic injection	Baseline, 1 and 2 months	(1) Increased blood flow was seen after probing whereas blood flow decreased after local anesthetic injection with vasoconstrictor. (2) Intrasulcular measurements were reproducible at 1 and 2 months	LDF is a non-invasive and reproducible tool capable of detecting alterations in blood flow in different time points
Dodson et al., 1994	Clinical	Assess value of intraoperative maxillary blood perfusion and describe pattern of blood flow changes during Le Fort I osteotomy	Orthognathic surgery	Intraoperative (10 s intervals)	Significant decrease of GBF of 64% during the fracture and mobilization compared to preoperative values	LDF is a feasible and valuable tool for measuring intraoperative maxillary blood flow dynamics
Hoke et al., 1994	Clinical	Develop a protocol for quantification of blood flow	Intraoral cavity	30 s intervals in 3 time points	High flows were in the buccal mucosa, vestibule and tongue. Medium flows were found in the attached gingiva	Intraoral tissue blood flow varies by site and be quantified non-invasively
Matheny and Johnson et al., 1993	Clinical	Determine the changes that occur in the gingival microcirculation during the development of experimental gingivitis	Gingivitis	2 time points	Significant decrease in gingival regional BF in gingivitis patients	Gingival microcirculation exhibited a dynamic change in response to the development and progression of gingivitis
Baab et al., et al., 1990	Clinical	Gingival blood flow and temperature were monitored continuously before and after cooling via a twin probe placed in the gingival sulcus	Periodontitis	5 min	Patients with periodontitis	Periodontitis patients showed significantly faster recovery of GBF than controls
Baab and Öberg 1987	Clinical	Study the acute effects of cigarette smoking on gingival blood flow	Tobacco smoking	5 min after smoking	Higher GBF during smoking	GBF increased significantly after smoking and stayed high during 5 min after
Baab et al., 1986	Clinical	Measure gingival blood flow after different stimuli: warm and cold water, pressure and biting force	Soft tissue measurements (free and attached gingiva, interdental papilla, alveolar mucosa)	Intervals of 30 s for water, 3 min for pressure and 1 min after biting force	No significant differences of blood flow after water stimuli (warm or cold), and pressure. Significant difference with occlusal biting force of 5 N	LDF is a non-invasive promising tool to study GBF

**Table 3:** Tissue perfusion with Laser speckle contrast speckle imaging (LSCI).

Author (year)	Spectral reflectance wavelength	Study design	Aim	Indication	Time evaluated	Results	Clinical relevance
Palombo et al., 2024	785 nm	Clinical	Compare early microvascular healing after palatal epithelialized gingival grafts (EGG) with sutures and hemostatic sponges (control group) or suturesless approach (test group)	Palatal epithelialized gingival grafts (EGG)	30 days	Test group showed earlier peak hyperemia at 7 days, with no significant blood flow differences between groups at any timepoint	The sutureless approach offers a viable alternative to the standard, with no significant microvascular differences
Vág and Mikecs. 2022	785 nm	Clinical	Compare the endothelium-dependent and independent vasodilation between men and women	Periodontitis	1 year	Endothelium-dependent vasodilation seems more pronounced in men than in women.	Might contribute to increased severity of periodontal disease in men
Mikecs et al., 2021	785 nm	Clinical	Investigate the mucogingival vasculature for functional analysis	Transient compression	2 time points (baseline and 30 s after compression)	Ischemia varied significantly from 0.26 08.76 mm in apico-mesial, apico-distal, or apical direction to compression	Individual variations in ischemic responses might explain unexpected outcomes
Amaral et al., 2020	638 nm	Pre-clinical	Differentiate the sound and osteoporotic maxilla and mandible bones in an *in vitro* model	Osteoporotic bone lesions	30 days	Osteoporotic tissue differentiates from healthy bone	Promising technique for assessment if osteoporotic lesions of alveolar bone
Fazekas et al., 2019a	785 nm	Clinical	Demonstrate the neovascularization pattern of a xenogenic collagen graft	Vestibuloplasty	98 days	Gingival microcirculation showed high regional variation.	LSCI is a suitable and reliable method for monitoring microcirculation in wound healing
Fazekas et al., 2019b	785 nm	Clinical	Investigate temporal and spatial blood flow patterns following vestibuloplasty using the collagen matrix	Vestibuloplasty	12 months	Neovascularization occurs from lateral and coronal surfaces and then spread concentrically to the center	Prolonged ischemia may be the reason for abundant scar formation
Molnar et al., 2019	785 nm	Clinical	Investigate correlation between clinical aspects of palatal wound healing	Connective tissue grafts	1 year	Reperfusion time and healing score were strongly correlated. In secondary healing intention sites postoperative blood flow was elevated with significant delay when compared to primary healing intention	LSCI is an objective method to assess individual wound healing and to predict quality of wound healing
Fazekas et al., 2018a	785 nm	Clinical	Monitoring blood circulation in the gingival area to determine optimal time for surgery for early implant placement	Immediate implant placement	19 months post extraction	Ischemic-hyperemia pattern occurs after tooth extraction from distal to base of the flap. Blood flow became stable after 6th weeks post-extraction	Understanding revascularization and stabilization of blood flow is key for optimal timing and decision making for second interventions individually
Fazekas et al., 2018b	785 nm	Clinical	Map spatial and temporal changes in gingival blood flow after transient compression	Transient gingival compression	5 s occlusion intervals with subsequent reperfusion for 20 min	Ischemia was greater coronal than apical to the occlusion line. Post occlusive hyperemia was observed in compressed and wider adjacent sites	Blood flow in the attached gingiva shows spatial differences. Blood circulation has an apicocoronal orientation of blood circulation
Gànti et al., 2018	765 nm	Clinical	Develop a technique proving the presence of spreading vasodilation in human keratinized gingiva	Application of nitric oxide (NO) in marginal gingiva of healthy patients	15 min	Higher doses induced significant elevation of gingival blood flow in all regions with apical prominence whereas lower doses showed increased flow only in the apical region	This mechanism may have a clinical importance for flap survival or wound healing
Molnar et al., 2018	785 nm	Clinical	Investigate the effect of factors inherent in oral mucosa measurement on intra-day and inter-day reliability	Gingival blood flow	1 week	Incidence angle influences mean blood flow without retraction. With retraction coefficients of variation were 8.3% intra-session and 10.5% inter-day. Coefficient of variation was 11.9% b alternating direct and indirect view	Good long- and short-term reliability regardless of lip retraction and indirect view
Molnar et al., 2017	785 nm	Clinical	Compare the effect of a xenogeneic collagen matrix to connective tissue grafts (CTG) on the microcirculation of the modified coronally advanced tunnel technique (MCAT)	Gingival recession coverage	6 months	Earlier blood flow recovery is strongly associated with earlier normalization of vascular permeability. Gender may influence postoperative circulation and inflammation	The LSCI method is suitable to capture the microcirculatory effect of the surgical intervention

**Table 4:** Wound healing assessment using a spectral imaging method.

Author (year)	Diagnostic tool	Spectral reflectance wavelength	Study design	Aim	Indication	Time evaluated	Results	Clinical relevance
Tang et al., 2025	Sidestream dark field (SDF)	520–525 nm	Clinical	Present a systematic framework for sublingual microcirculation monitoring is proposed, combining optical imaging and automated vessel analysis.	Novel parallel probe-based (PPsSDF) for sublingual monitoring	1 time point	A novel sublingual imaging system is developed and validated, featuring a front-end probe aligned parallel to the underside of the tongue to minimize tissue pressure.	The proposed PP-SDF device and automated analysis framework show strong performance and hold promise for widespread clinical use in microcirculation monitoring.
Cusack et al., 2025	Sidestream dark field (SDF)	Not specified	Clinical	Measure sublingual oral mucosa capillary flow and density	Septic subjetcs	4 time points (baseline, 15 min, 60 min and 28 days after albumin bolus administration	The albumin group showed significant improvements in microvascular density and activity at 15 and 60 min the control group showed no significant change.	A 20% albumin bolus markedly improves microcirculation in fluid-responsive patients with septic shock.
Heitzer et al., 2025	Laser Doppler spectrophotometry Near infrared spectroscopy (NIRS)	710 nm–775 nm	Pre-clinical	Compare the hemostyptic properties and gingival healing after tooth extraction in rodent under rivaroxaban therapy over 10 days	Hemostyptic properties (mean oxygen saturation and relative hemoglobin) between novel polyurethane based adhesive VIVO and gelatin sponge (GESP)	1 time point	Increase mean oxygen saturation for both treatments. GESP showed significant increase in relative hemoglobin	VIVO demonstrated effective hemostasis and favorable gingival healing following tooth extraction under continuous rivaroxaban therapy
Katz et al., 2024	Laser Doppler spectrophotometry	Not specified	Clinical	Evaluate peri-implant tissue oxygen saturation (SO2),and relative amount in hemoglobin (rHb) in implants placed in pristine bone, avascular, and microvascular grafts	Perfusion in different bone types in dental implants	1 time point	No differences found in gingival blood flow in either pristine or avascular bone or microvascular grafts	Perfusion was similar between implants in native and augmented sites, though those in avascular or microvascular grafts showed increased peri-implant inflammation
Ooms et al. 2023	Laser light Doppler spectoscopy	830 nm and 30 mW	Clinical	Monitoring postoperative flap perfusion in oral microvascular reconstruction	Flap perfusion monitoring	48 h	This method can detect vascular compromise	Reliable method for postoperative flap monitoring
Preidl 2021	Laser Doppler spectrophotometer	(830 nm and 30 mW) and white light (20 W, 500–800 nm)	Clinical	Detect perfusion in porcine collagen membranes (CMs) within an open wound situation compared to free gingival grafts (FGG).	Vestibuloplasty	Intraoperatively and post operative until 90 days	No significant differences between CM and FGG. Blood flow increased until day 10	Perfusion pattern showed similar behavior instinctively of the biological differences
Sekhar Prasanth et al., 2013	Diffuse reflectance spectral imaging (DRSI)	620 nm	Clinical	Map gingival inflammation in periodontal disease *in vivo*	Periodontitis	1 time point visit	Mild and moderate inflammatory tissues were observed from healthy with 92% and 83%, sensitivity and 93% and 96% specificity, respectively	High diagnostic accuracy is used to detect underlying inflammation
Bodo et al., 2013	Near infrared spectroscopy (NIRS)	N/A	Pre-clinical	Quantify tissue oxygenation of soft tissue healing	Bone healing fractures comparing wound treatment: salmon fibrinogen/thrombin, bone filler matrix, bovine collagen, porcine fibrinogen/thrombin	Immediate post-surgical until 3 weeks	Soft tissue healing did not differ significantly. However, regional oxygenation of wounds treated with salmon fibrinogen/thrombin showed slightly different time trends.	Non-invasive quantification of soft tissue healing supports the aims for evidenced-based medicine. This tool shows potential toward understanding the role of early revascularization in healing
Milstein et al., 2009	Sidestream dark field (SDF)	530 nm	Pre-clinical	Assess the acute effects of chemotherapy on gingival microcirculation	Gingiva	2 time points visits 30 min apart	Capillary density increases in direct relationship to chemotherapy dosage	Functional response of microcirculation plays a role in contribution to oral communications and oral tumor treatment
Zakian et al., 2008	Diffuse reflection spectroscopy	460–615 nm	Clinical	Demonstrate that gingival disease progression evaluated with spectral imaging can correlate to Gingival index (GI) scores	Periodontal disease	6 weeks	Quantification of gingival inflammation changes correlates significantly with GI scores	Development of functional imaging methods for disease detection and diagnosis
Lindeboom et al., 2006	Orthogonal spectral polarization (OPS)	548 nm	Clinical	Evaluate intra observer agreement in the assessment of gingival capillary density	Gingival buccal surface of periodontal healthy patients	1 time point	High inter-observer agreement and intraclass correlation	OPS is an immediate, reproducible and acceptable method to quantify assessment of gingival microcirculation

**Table 5:** Wound healing assessment by ultrasound (US) color flow.

Author (year)	Imaging frequency	Study design	Aim	Indication	Time evaluated	Results	Clinical relevance
Tavelli et al., 2025	24 MHz	Clinical	Assess Doppler tissue perfusion at dental implant sites augmented with connective tissue grafts	Soft tissue augmentation in dental implants using coronally advanced flaps (CAF) or tunnel technique (TUN)	12 months	Mid-facial color and power changes were comparable between CAF and TUN, with significant differences noted only at interproximal sites	Noted at the interproximal sites, early perfusion was positively associated with both clinical and volumetric outcomes at the 12-month follow-up
Samal et al., 2024	24 MHz	Pre-clinical	To quantify inflammation by detecting tissue dimensional and perfusion changes.	Gingival inflammation after bacterial inoculation	4 weeks	Color flow velocity was significantly elevated for 6 weeks, except in male 2nd premolar at week 2. Power changes were significant only in 1st molar, with effects influenced by tooth type and sex	Periodontal tissue thickness and color flow velocity increased in response to bacterial inoculation
Sirinirund et al., 2023	24 MHz	Clinical	Evaluate soft and hard tissues changes during GBR healing and after implant function	Guided bone regeneration	5 months	Blood flow increased at 1 and 2.5 months after surgery and decreased at 5 months	Identify early healing deviations and addresses to improve the clinical workflow and quality of patient care
Barootchi et al., 2022	24 MHz	Clinical	Assess if tissue perfusion correlates with clinical diagnosis	Implants in health and disease	1 visit	US quantified color velocity (CV) and power (CP) directly correlates with clinical diagnosis	First study to prove that US color flow can be applicable in the diagnosis of peri-implant health and disease. Is a valuable to evaluate degree of inflammation
Siqueira et al., 2021	24 MHz	Clinical	Explore feasibility of US for clinical imaging	Peri-implantitis and 2nd stage implant surgery	3 months	Blood flow imaging showed a dynamic range with different degree of inflammation	Functional blood flow imaging may be useful to estimate the extent and severity of inflammation
Tavelli et al., 2021	24 MHz	Clinical	Describe the application of US for evaluation blood flow at implant and palatal donor sites	Soft tissue augmentation with the connective tissue graft (CTG)	12 months	US captures and estimated tissue perfusion at different times points during wound healing	US power Doppler is a non-invasive and suitable tool for identifying conditions with abnormal blood flow and subclinical inflammation
Chan and Kripfgans 2020	24 MHz	Clinical	Propose ultrasound imaging as a potential solution in the diagnosis of peri-implant disease	Peri-implant disease	1 time point	Ultrasound can provide functional images (color flow images) that may be useful for evaluating the degree of peri-implant tissue inflammation	High-frequency US could be a cross-sectional imaging modality in adjunct to radiographs for diagnosing imminent peri-implant disease
Izetti et al., 2020	70 MHz	Clinical	Preliminary study to characterize normal oral mucosa tissues using ultra high frequency ultrasound (UHFUS)	Oral mucosas	1 time point	Gingiva: Moderately vascularized with several low-velocity vessels visible in Doppler mode. Lip: Moderate vascularity with increased flow near minor salivary glands; terminal labial artery branches occasionally appear as pulsing round structures.	A potential role for intraoral UHFUS in enhancing assessment, diagnosis, and management of oral mucosal conditions.
Bodo et al., 2013	9.5 MHz	Pre-clinical	Quantify blood flow of soft tissue healing	Bone healing fractures comparing wound treatment: salmon fibrinogen/thrombin, bone filler matrix, bovine collagen, porcine fibrinogen/thrombin	Immediate post-surgical until 3 weeks	No significant differences for wound treatment in inflammation severity and blood flow. Salmon fibrinogen/thrombin showed rapid revascularization.	Non-invasive methods may be useful for human clinical application and needs to be incorporated in wound healing index
Tikku ete al. 2010	9 MHz	Clinical	Evaluate effectiveness of US color Doppler in monitoring the post-surgical healing	Periapical lesions of endodontic origin	6 months	US anatomical and functional modes showed significantly higher than conventional radiography in detecting tissue changes	US Color flow has the potential to monitoring of post-surgical healing

**Table 6:** Other imaging techniques for oral perfusion assessment.

Author (year)	Diagnostic tool	Study design	Aim	Indication	Time evaluated	Results	Clinical relevance
Takeda et al., 2023	Capillaroscopy	Pre-clinical	Examine gingival capillaries and the influence of diabetes on gingival microcirculation	Periodontitis	1 time point	Gingival microcirculation was not found to be associated with periodontal parameters, but the presence of capillary morphology was higher in diabetes.	Morphological abnormalities of gingival capillaries were oral manifestations of diabetes
Townsend 2022	Laser perfusion, Video Capillaroscopy and OCT	Clinical	Measure changes in arteriolar and venular capillary flow and structure in the gingival tissues during plaque gingival inflammation	Periodontitis	3 weeks	Reduced capillary flow associated with the development of venular capillaries	Microvascular imaging in gingival tissue could help to understand why some people develop periodontal breakdown
Yilmaz and Atlas 2021	Videocapillaroscopy	Clinical	Evaluate microvascular alterations (capillary loop visibility, capillary orientation to surface, microhemorrhages, capillary density and tortuosity) in patients with gestational diabetes mellitus (GDM)	Maxillary anterior masticatory or gingival mucosa.	1 time point	Capillary density was increased in gestational DM. Higher tortuosity scores are seen in healthy non pregnant subjects	There is an impact on gingival microcirculatory changes in pregnancy and GDM
Le et al., 2018	Optical coherence tomography (OCT) and optical coherence angiography (OCTA)	Clinical	Propose a 3D technique to perform *in situ* imaging on human gingiva	Labial gingival tissues	1 time point	Significant structural and vascular differences between thin and thick biotypes	OCT/OCTA is feasible in quantifying gingival biotypes and inflammation severity
Bastos et al., 2016	Real time optical vascular imaging technique (RTOVI)	Clinical	To show a new technique to detect early signs of oral cancer in the gingival microvasculature for number of capillaries, capillary area (CA), total capillary area (TCA) and aspect ratio (AR)	Oral squamous cell carcinoma (OSCC) and oral potentially malignant disorders (OPMD)	1 time point	Significant differences highlighting regional microvascular differences. TCA, APC, and AR showed no significant differences in healthy gingival tissue. The mean capillary count was higher than previously reported for the same oral mucosal location.	Mean values of CA, TCA and RA may be a reliable threshold values to compare healthy, inflammatory and mitotic lesions
Janovszky et al., 2014	Intravital Videomicroscopy (IVM)	Pre-clinical	Investigate zoledronate induced microcirculatory reactions in mandibular periosteum in comparison with the tibia with bisphosphonate induced medication	Tooth extraction	9 weeks	Use of zoledronate induced significantly higher degree of periosteal leukocyte rolling and adhesion in the mandibular postcapillary venules (in extracted and intact sites)	This model may contribute to the development of strategies to counteract bisphosphonate induced side effects
Scardina et al., 2014	Video capillaroscopy	Clinical	Evaluate biological microvascular response to orthodontic forces	Orthodontics forces	12 months	Capillary density increases significantly directly proportionate to application time of the orthodontic device	*in vivo* evaluation and quantification of microcirculatory changes and detecting subclinical changes in angiogenesis
Takano et al., 2010	Narrowband imaging (NBI)	Clinical	Analyze and describe intracapillary loops (IPCL)	Oral neroplastic lesions	1 time point	Non-neoplastic lesions showed mild capillary changes, while neoplastic had irregularly distributed loops with varied shapes	NBI may aid early diagnosis of oral squamous cell carcinoma and help define resection margins by visualizing microvascular patterns

### 3.2 Risk of bias assessment

**Figure 2:**
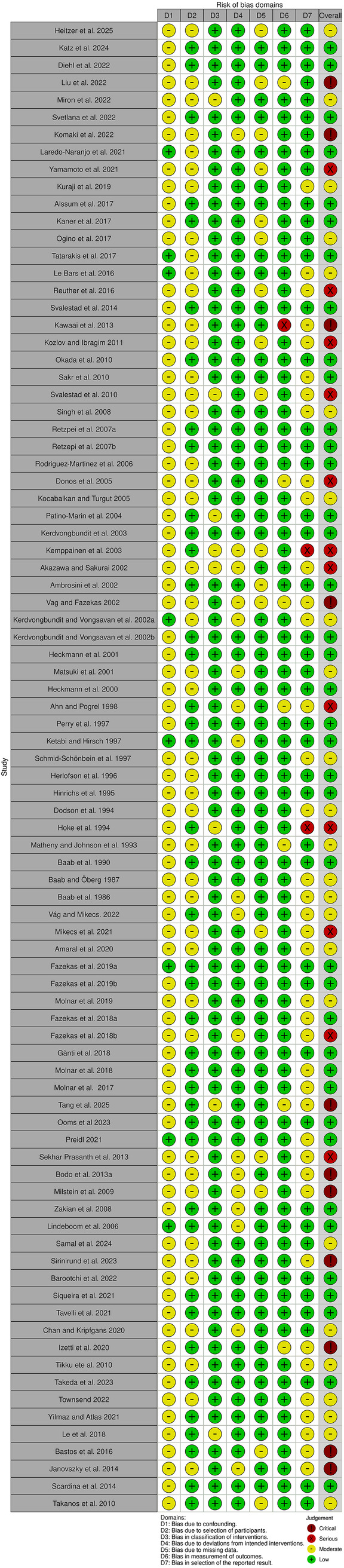
Diagram illustrating risk-of-bias assessment of the non-RCTs using the ROBINS-I tool.

**Figure 3:**
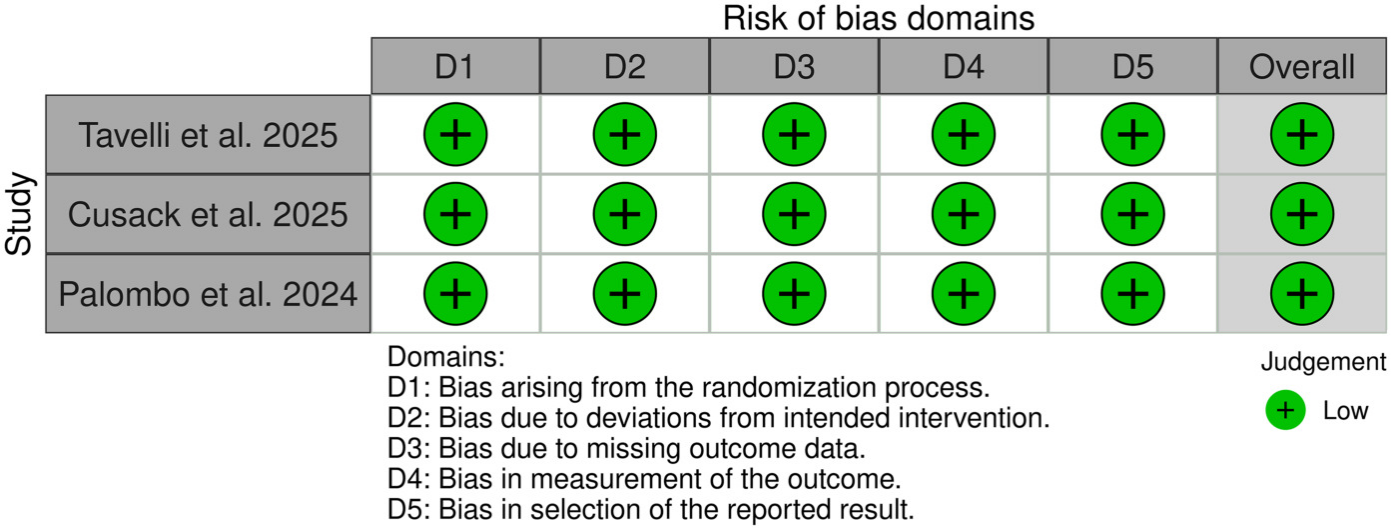
Diagram illustrating risk-of-bias assessment of the selected RCTs using the RoB-2.

Among the 91 included studies, 11 were preclinical, 77 were clinical non-RCT studies (Figure 2), and 3 were clinical RCTs (Figure 3). Of the non-RCT studies, 40 were assessed as having a “low” overall risk of bias, 24 had a “moderate” risk, 12 were rated as “serious”, and 10 were deemed to have a “critical” risk of bias (Supplementary Table S1). All three RCTs were evaluated as having a “low” risk of bias. These findings are visualized in Figures 2, 3 (39).

### 3.3 Laser Doppler flowmetry (LDF)

Laser Doppler Flowmetry is based on the frequency shift of the backscattered laser light from moving objects (Doppler shift principle) (40–43). When an infrared laser is reflected by or scattered off of red blood cells (RBC), a frequency change is seen (12, 25, 40, 42, 44). The system uses a probe with a focal distance of less than or equal to 3 mm. Probe placement is in a perpendicular orientation to the area of interest (43, 44) (Table 2). A total of 49 studies were identified related to LDF. It was used to assess microvascular dynamics of the gingiva (45–54), several bone types (55), periodontal and peri-implant disease (44, 56–60), flap design (12, 15, 25, 61), periodontal tissues exposed to occlusal trauma (40), occlusal loading in denture-supported tissues (62–65), orthodontic treatment (42), orthognathic surgery (24), the effect of hyperbaric oxygen therapy (HBOT) (43, 66), burning mouth syndrome (BMS) (67), septic shock (68), periodontal tissues reaction during prosthetic rehabilitation (69), tooth brushing (70, 71), the effect of toothpaste detergent (72), the effect of temperature stimuli (52, 73, 74), perfusion characterization after transient compression (6, 75), hemostyptic properties and gingival healing (76) the effect of local anesthetic (77–79), the effect of dexmedetomidine (80), early vascular response (12, 15, 17, 25, 29, 81), smoking (82, 83), wound healing in type 2 diabetes patients (84), and gingival tissue regeneration (85) (Table 2). LDF is easy to perform, non-contact, and economical. The main disadvantages of LDF are its sensitivity to light, artificial motion, pressure, probe angle. Other drawbacks include (1) arbitrary perfusion unit presented, (2) lack of anatomical images, and (3) superficial penetration depth (up to 1 mm) (40, 41).

### 3.4 Laser speckle contrast imaging (LSCI)

**Figure 4:**
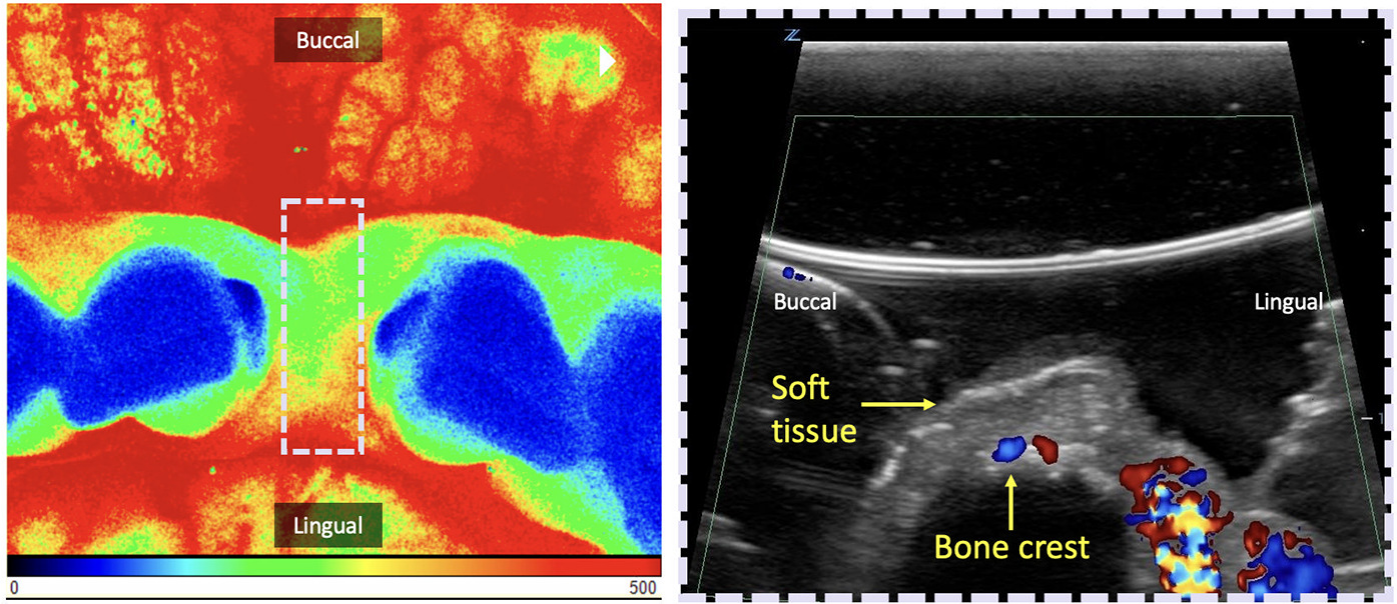
Demonstration of two perfusion imaging techniques of the edentulous crest. Both can be used in the occlusal view for pre-assessment of future implant placement in the anterior mandible. *Left:* Laser Speckle Contrast Imaging (LCSI). Here, blood flow is expressed through Laser Speckle Perfusion Units (LSPU) and depicted as false color images: blue-cyan (low perfusion), green-yellow (moderate perfusion), orange-red (high perfusion). The region of interest (ROI) delimited by the dashed white rectangle is the location of the ultrasound shown on the right. *Right:* Cross-sectional B-mode ultrasound image with color flow in velocity mode. Color velocity images display hues of blue to red, which correspond to the detected blood flow velocity. Changes in this velocity potentially serve as a quantitative indicator for the degree of inflammation. In this case, in both techniques, the presence of high tissue perfusion can be seen on the lingual surface and low to medium perfusion mid-occlusal.

LSCI quantifies the magnitude of the superficial tissue perfusion by analyzing the speckle pattern of scattered light using an invisible laser that is scanned over a 2D region of interest (wavelengths close to 785 nm) (10, 27). This technique estimates the velocity of the moving targets, in this case RBCs, across a 2D surface and therefore also incorporates the gingival vascular density and thus its fractional blood volume. Blood flow velocity distributions are shown as relative color encoded perfusion units (LSPU): blue-cyan (low perfusion), green-yellow (moderate perfusion), and orange-red (high perfusion) (Figure 4) (11, 26, 31, 32).

A total of 12 studies were identified related to LSCI (6, 10, 11, 18, 26, 28, 31–33, 69, 86, 87). In them, LSCI was used for evaluating gingival perfusion after transient compression of osteoporotic lesions, after vestibuloplasty, after immediate implant placement, in response to nitric oxide, factors influencing the readings, compare vasodilation between men and women to evaluate the increase in the severity of periodontitis (10, 86) and after soft tissue augmentation procedures (18) (Table 3). Two studies validated the reproducibility of this technique (10, 32, 33). A recent randomized clinical trial (87) evaluated early microvascular healing after palatal epithelialized gingival grafts (ECG) harvesting using traditional hemostasis vs. a sutureless approach, assessed by LSCI at 7, 14, and 30 days. The sutureless group showed faster hyperemia resolution and a 13-min shorter surgical time, with no significant differences in perfusion, bleeding, or analgesic use, supporting it as a viable alternative. Major advantages are: (1) large field of view that can cover few teeth and (2) anatomical images included, allowing knowledge of the spatial distribution of the circulation. LSCI is limited by the need for direct line-of-sight, restricting access to areas like the molars. It also has shallow penetration (∼1 mm), is sensitive to working distance, patient movement, gingival temperature, and measurement angle—especially in curved regions like the maxilla and mandible, which can affect blood flow readings (28) (Table 3).

### 3.5 Spectral imaging methods

Spectral methods have been identified for evaluating periodontal tissue perfusion in 7 articles. The light source can be either ultraviolet-visible (UV-VIS), Diffuse Reflectance Spectroscopy, DRS) or near-infrared (Near Infrared Spectroscopy, NIRS). The mechanisms by which the interaction of light and matter can be quantified include the well-established Diffuse Reflectance Spectroscopy (DRS), Orthogonal Spectral Polarization (OPS), and most recently Sidestream Dark Field (SDF) (30, 88–91) (Table 4). The reflected light in at different wavelengths after it interacts with the sample is quantified and reported, in our case, oxygen saturation, and hemoglobin concentration. Laser Doppler Spectroscopy (LDS) integrates laser Doppler and visible light spectroscopy to assess soft tissue grafts after vestibuloplasty at a 2 mm tissue depth (90). It uses continuous-wave laser light (830 nm, 30 nW) and white light (20 W, 500–800 nm), with real-time signal detection via a single optical probe connected to a computer. Measurements include blood flow velocity and flow (AU), derived from Doppler shifts, tissue oxygen saturation (SO₂%) based on light absorption/reflection, and relative hemoglobin concentration [rHb], independent of vessel density or size (90). Laser Doppler flowmetry and tissue spectrophotometry (LDF-TS) (55, 76) have also been used to monitor microsurgical flaps at extraction sites (76) and peri-implant tissues (55), providing quantified perfusion data including SO₂ (%), rHb (AU), and blood flow (AU). This system's intraoral use is made possible by a compact 5 × 2 mm probe with 1 mm depth, specifically designed for such applications (55).

Near Infrared Spectroscopy (NIRS) offers significantly deeper soft tissue penetration (∼25 mm) (20). In contrast, UV/Vis spectroscopy assesses corneocyte levels at a much shallower depth (∼430 µm) in skin within preclinical models (92). Both, NIRS and UV/Vis spectroscopy quantify corneocyte amounts but differ in wavelength ranges and measurement approaches (92). NIRS in the near-infrared and UV/Vis in the visible spectrum. As a result, pseudo-absorption values are not directly comparable due to differing interactions with skin components (92). Changes in tissue oxygenation may be an indicator of tissue inflammation (20). Tissue hypoxia was defined when oxygen tension is <40 mmHg. DRS has shown a significant correlation between the Gingival Index and the level of gingival inflammation (89, 91). Additionally, *in vivo* near-infrared (NIR) fluorescent imaging scan can be acquired with ultrafocus magnification via 360° rotation at 0.75° increments (0.3 s/°) and requires fluorescent dyes (76). Fluorescence imaging was performed first using a laser and filter system (excitation: 710 nm, emission: 775 nm), with data captured by a cooled CCD camera and reconstructed into 2D images (76). Some limitations of this technique are the lack of laterality (limited field of view) and the fact that NIRS does not distinguish between hemoglobin and myoglobin (20). Other limitations include the possibility of cross-contamination, optical probe size, sensitivity to optical probe position, and pressure (91) (Table 4). Due to the promising capabilities of spectral imaging in quantifying physiological tissue components, a recent study introduced an RGBIR (Red, Green, Blue, and Infrared) image sensor designed to capture spectral information from both oral and skin tissues (93). Tissue optical including chromophore concentrations and scattering characteristics, is essential for modeling light behavior and optimizing device design. Due to variability between individuals and tissue sites, real-time measurement is often necessary for personalized care (94). Red wavelengths typically range from approximately 620 to 740 nm, green from 520 to 570 nm, and blue from 440 to 470 nm. The objective was to estimate chromophore concentrations. Raw pixel values were calibrated to reflectance and then mapped to a diffusion model-based lookup table, linking tissue reflectance to melanin, oxygen saturation, and water content (93). Initial benchtop experiments demonstrated chromophore changes corresponding to an applied finger occlusion. The technique was later applied to intraoral imaging, where reflectance changes were associated with areas of erythema (93). Additionally, a color polarization camera (RGB) was utilized to quantify blood volume and tissue oxygen saturation in both superficial and deep layers of the skin (95). This system captures co- and cross-polarized RGB images within a single acquisition frame. Polarization data enables the differentiation of skin layers, while RGB color information serves as input for a neural network model that predicts tissue blood volume and oxygen saturation. Findings demonstrated *in vivo* differentiation based on age and skin tone among human subjects (95).

Orthogonal Spectral Polarization (OPS) uses linearly polarized green light (548 µm) to illuminate the sample. The reflected depolarized light is then received by a sensor in orthogonal direction to the emitter. It can measure capillary blood flow, vessel diameter, and vessel density (96). The system measures tissue perfusion using two types of optical probes: an attached surface probe and an unattached surface probe. The attached probe, which assesses perfusion at a depth of 3 mm, was secured with four sutures at the center of the radial forearm free flap (96) and about 1 cm from the perforator vessel in the anterolateral thigh flap (ALTF). The unattached probe, capable of measuring at depths of 2 mm and 8 mm, was manually positioned parallel to the attached probe during each reading (96). The advantage of OPS over other spectral techniques its sensitivity to capillary blood flow, vessel diameter, and vessel density. The latter has been used to estimate tissue perfusion (96). Limitations include optical probe positioning, patient movement, and lack of access to the interdental gingiva (88).

Insufficient image sharpness in OPS led to the development of Sidestream Dark Field (SDF) imaging, a non-invasive technique used to quantify capillary density in the mucosal microcirculation (97). SDF specifically visualizes the flow of red blood cells through small vessels such as capillaries, using green light at a wavelength of 530 nm, which is absorbed by hemoglobin to enhance contrast and enable clear visualization of microvascular flow (30). Milstein et al. described a handheld video microscope based on SDF technology, which employs concentrically arranged light-emitting diodes (LEDs) around a central light guide. These LEDs emit light at wavelengths that are absorbed by both oxy- and deoxyhemoglobin, allowing detailed imaging of red blood cell movement (30). To overcome limitations of traditional vertical-capture SDF systems, Tang et al. introduced a novel parallel probe-based SDF (PP-SDF) device, offering improved portability and usability (98). Clinical studies have demonstrated this device's strong performance and its potential for future applications in real-time microcirculation monitoring (98). Furthermore, a recent randomized controlled trial by Cusack et al. assessed the microcirculatory impact of fluid resuscitation in septic shock and found that a 20% albumin bolus significantly improved microvascular parameters compared to crystalloids, despite similar macro hemodynamic measures, highlighting the concept of hemodynamic incoherence (99). The advantages of SDF include reduced image contamination from tissue surface reflections and a wider imaging field; however, limitations such as low image resolution and contrast still remain (30).

### 3.6 Ultrasound (US)

Ultrasound is a cross-sectional imaging modality based on the reflection and scattering of sound by the interaction of emitted acoustic waves with tissues (19, 34, 100). For tissue perfusion evaluation, flow modalities, e.g., Color Velocity (CV), and Color Power (CP) have been used. The grayscale (B-mode) display is overlaid with color pixels in which blood flow velocities are displayed that exceed the (noise/wall) filter threshold. Flowing red blood cells scatter the incident acoustic wave and produce a scattered signal that changes in the radio frequency phase if the flow direction is non-perpendicular to the ultrasound beam. CV images display red and blue colors, which correspond to the orientation of the detected blood flow velocity (speed) with respect to the ultrasound beam direction. CP is displayed as a single hue of red, that is quantitatively or qualitatively proportional to the amount of moving blood within the ultrasound resolution cell associated with a given pixel. Additionally, CV and CP can, as a surrogate, describe dynamic blood flow, and thus tissue perfusion, variation as an indicator of the degree of inflammation, correlation with clinical disease diagnoses, and healing monitoring (19, 20, 22, 34, 100–104) (Table 5, Figure 4). A recent RCT (103), compared tissue perfusion at implant sites treated with connective tissue grafts using either a coronally advanced flap (CAF) or tunnel technique (TUN). While mid-facial perfusion showed no significant difference, TUN demonstrated higher interproximal perfusion and early graft vascularization. Early perfusion was associated with improved soft-tissue outcomes at 12 months, highlighting Doppler ultrasonography as a valuable tool for monitoring healing. In addition, a preclinical study (105), assessed inflammation by measuring tissue thickness and perfusion changes in mini-pigs following bacterial inoculation. Soft tissue thickness and color flow velocity significantly increased post-inoculation across most teeth and time points, with variations by tooth type and sex. These changes suggest a correlation between bacterial challenge and periodontal inflammation, though tooth eruption may have influenced results. Limitations of this imaging technology are the reliance on a (gel) coupling medium, potential user dependency, sensitivity to probe pressure over tissues, scanning angulation, and access (Table 5).

### 3.7 Other imaging techniques

Intravital Video Microscopy (IVM) (106) is mainly used in preclinical studies because of the use of fluorescence dye and and maximal sample stability. A study demonstrated how zoledronic acid may induce microcirculatory inflammatory reactions in the mandibular periosteum in contrast to long bones (tibia). In addition, it was used to examine gingival capillaries during plaque-induced gingival inflammation and to evaluate for the influence of diabetes on gingival microcirculation in pre-clinical and clinical studies (107, 108) (Table 6). The advantage of IVM is that it quantifies microcirculatory parameters of red blood cell velocity (RBCV, μm/s). This takes place offline by inter frame analysis of the recorded images. Limitations of this technique include the need of a fluorescent dye and only superficial penetration (106) (Table 6). Another example is Oral Videocapillaroscopy using a fiber optic probe with high color resolution, a focal spot size of 1.8 mm, and variable magnification (10–1,000×) (109) (Table 6). On the other hand, Videocapillaroscopy was conducted using a computerized video microscope system with a 200× optical probe, and a varying focal spot of (0–2 mm). All assessments were performed by the same operator in the morning under consistent lighting conditions (110). Limitations of this technique is that it only visualizes capillary apices in gingiva, limiting assessment of full loop structure, and potential microvascular changes from overweight status (110).

Optical coherence tomography (OCT) with OCT angiography (OCTA) is a noninvasive 3D imaging method used to assess gingival tissue *in situ* (111). OCT/OCTA provides detailed structural and vascular information up to 2 mm deep for improved periodontal evaluation. The hand-held OCT probe allows movement in three translational and one rotational direction for precise positioning, with stabilization provided by a bite bar (111). The system uses a swept-source OCT with a 1,310 nm wavelength and 110 nm bandwidth, offering 7 μm axial resolution. This study employed a lightweight probe mounted on a kinetic arm for accurate gingival scanning. Lee et al. highlighted significant structural and vascular differences between thick and thin gingival biotypes, as well as distinct vascular patterns in gingival inflammation. OCT/OCTA is effective for quantifying these biotypic attributes and inflammation severity (111). Limitations of OCT/OCTA include issues with vascular leakage, slow-flow lesions, artifacts, patient stillness, and device variability (111).

Additionally, Bastos and Cook 2016 introduced real-time optical vascular imaging (RTOVI), which detects early signs of oral cancer by observing the gingival microvasculature with green light (112). RTOVI visualizes tissue function and behavior, assessing microvascular features like capillary count, area, and aspect ratio (112). The system uses a specific wavelength (550 ± 10 nm) to optimize red blood cell imaging, combining transmission and reflection techniques. RTOVI enables detection of individual red blood cells (RBCs) in the microvasculature, clearly delineating its structure. RTOVI utilizes a field of view (FOV) of 0.51 × 0.51 mm, with a pixel resolution of 0.35 × 0.35 µm. The study found a higher mean capillary count (45.06 per mm^2^) compared to previous studies, suggesting that RTOVI, alongside image analysis methods, could serve as a reliable tool for monitoring microvascular changes and exploring inflammatory or precancerous lesions (112). Limitations for this technique include low contrast and not enough resolution. Takano et al. (113) investigated intrapapillary capillary loops (IPCLs), key indicators of early oral neoplastic changes, using a narrowband imaging (NBI) system. NBI enhances visualization of mucosal surfaces and microvasculature by employing optical filters that narrow light bandwidths. When combined with magnification, it improves early cancer detection by highlighting vascular features such as IPCLs. Originally developed for the pharynx and esophagus, NBI has since been adapted for oral cavity assessment. The system uses rotating RGB filters (500, 445, and 415 nm, each with ∼30 nm bandwidth), enabling superficial tissue penetration (170–240 μm) for detailed vascular imaging. However, its effectiveness is limited in areas of hyperplasia (e.g., leukoplakia) and mucosal erosion, where IPCL structures may be obscured or destroyed.

## 4 Discussion

### 4.1 General discussion

A few technologies have been identified to study oral vasculature non-invasively in this review, including optical as well as ultrasound based methods (Tables 1–6). They were used to detect and quantify microcirculatory changes resulting from oral diseases, exterior stimuli, and surgical interventions. IVM is among the first developed imaging technology for evaluating microcirculation. It is primarily used for preclinical studies. VC was then developed for easily accessible locations. Technology advancement leads to OPS and soon later SDF. Compared to VC, OPS has higher contrast, is user-friendly, and costs less. SDF enjoys higher image sharpness and contrast than OPS. These 4 methods can provide morphologic (capillary size/diameter) as well as functional (blood flow velocity in mm/s) evaluation of capillaries. DRS and NIRS are another category of imaging technology, generally used for estimating oxygenation and Hb concentration. Last, LDF provides arbitrary perfusion units based on Doppler phase shift without spatial/anatomical information. LSCI also provides arbitrary perfusion units based on speckle pattern changes with a larger field of view and spatial/anatomical information.

### 4.2 Clinical impact of studying oral microcirculation

Previous studies observed an ischemic-reperfusion pattern of gingival blood flow with faster recovery rates in minimally invasive flaps when compared to extensive incisions and flap design (12, 15, 17, 25, 29, 81). Faster blood flow recovery is strongly associated with earlier normalization of vascular permeability, and periosteal perfusion significantly influences bone healing and determines the prognosis of soft tissue survival (109). In addition, gingival perfusion changes is suggested as an indicator of disease severity (17, 19, 33, 34, 44, 58–60). Imaging technologies that objectively monitor healing in real time, using non-invasive methods, are urgently needed for diagnostic purposes and to inform management strategies. This could potentially eliminate human bias and facilitate integration of artificial intelligence (AI) in perfusion analysis. Their relevant clinical applications span from pre-surgical to intraoperative and post-surgical assessments. Pre-surgically, it is beneficial to obtain three-dimensional maps that visualize the distribution of microcirculation for preventing vascular complications. Perfusion evaluation can document changes in gingival blood flow and assess response to treatment. The integrity of collateral microcirculation in the gingiva is vital for surgical predictability, healing speed, patient morbidity, and wound closure probability (1). Vascular flow alterations may provide insight into a better understanding of tissue healing capacities, which would translate into better clinical performance (10, 12, 15, 19, 20, 34, 40, 42). Therefore, understanding the dynamics of oral microvasculature during incision tracing and flap design may result in optimal vascular reorganization and wound healing (1, 10, 11, 26).

Reliability is key for use of these technologies. Temporal fluctuation of flow does not necessarily reflect myogenic vasomotion; in fact, it can have multiple causes (6, 11, 18). Flow sensitivity may be influenced by systemic conditions such as diabetes, arterial hypertension, and behavioral factors, such as smoking (17, 26, 27, 41, 42, 106). Changes in gingival microcirculation can be related to physiological events synchronized to neurodynamic activity, ventilatory influences, gravitational effects, and arterial and venous pressure (31, 32, 42). In addition, other studies have investigated the high temporal variation of gingival blood flow (Tables 2–5) that may be related to physiological factors such as blood pressure (59), the effect of temperature (52, 73, 74), mechanical stimuli (10), gingival inflammation (17, 19, 22, 31–34, 44, 60), circadian rhythm, occlusion forces (40, 69), orthodontic forces (42), and tooth brushing (71). These factors should be carefully considered and evaluated to improve the reliability of using these technologies as a research tool.

### 4.3 Features of a desirable device for quantifying microcirculation

An ideal device for microcirculation quantification would possess features that relate to probe/device geometry, the relationship to actual physiological blood flow/perfusion, measurement accuracy and precision, data recording time and data presentation, as well as infection control requirements. In addition, microcirculation maps (surface and/or cross-sectional) should be generated with high spatial resolution (∼100 µm) for better definition. Furthermore, the device should be portable, affordable, and have minimal environmental constraints.

### 4.4 Available technologies comparison

A summary on the advantages and limitations of the included technologies is included in Table 7. Single-point LDF was developed in 1970s and is most frequently studied for *in vivo* periodontal tissue perfusion. It provides arbitrary unit for tissue perfusion in a focal region of ∼1 mm^3^. LSCI quantifies the magnitude of tissue perfusion with a color scale superimposed on the anatomic structure in a larger field of view when, compared to single-point non-anatomical LDF (28, 31–33). Common limitations of LDF and LSCI include limited depth of penetration, making both only suitable for superficial circulation evaluation and provision of arbitrary perfusion units that require a reference for comparison, such as the baseline or contralateral measurements (6, 10, 11, 18, 26–28, 31–33, 41) (Table 3). The field of view provides mesio-distal frame that can vary from the recording of a wider area of interest provides advantages of LSCI over LDF, DRS, OPS, and to US because it gives insight into adjacent tissues. This compensates for some of the limitations compared to other techniques as it offers additional anatomical information.

**Table 7:** Advantages and limitations of the perfusion assessment techniques.

Perfusion technique	Laser Doppler flowmetry (LDF)	Laser speckle contrast imaging (LCSI)	Spectral imaging methods (LDS, DRSI, NIRS)	Ultrasound (US)	Sidestream dark field (SDF) Orthogonal spectral polarization (OSP) Real-time optical vascular imaging (RTOVI)	Intravital microscopy (IVM), Video capillaroscopy, Optical coherence tomography angiography (OCTA), Narrow band imaging (NBI)
Advantages	Non-contact Relatively easy to perform	Non-contact Wide field of view (can up to cover few teeth) Provide anatomical images and perfusion data	Allow evaluation of tissue composition/property, such as oxygenation, hemoglobin concentration, and water content	Cross-sectional (good penetration depth than the other optical-based techniques) Provide anatomical images, vessel distribution, perfusion direction, and velocity/volume of blood flow	Non-contact High spatial resolution Visualize capillaries True blood velocity measurements	Non-contact High spatial resolution Visualize capillaries True blood velocity measurements
Disadvantages	Arbitrary tissue perfusion units provided No anatomical images Limited penetration depth	Arbitrary tissue perfusion units provided Limited penetration depth Large sensor (molars are hard to image)	Limited penetration depth Lower sensitivity Generally, no anatomical images provided	Surrogate tissue perfusion Need contact via coupling agent	Limited penetration depth Sensitive to motion Limited field of view	Very limited penetration depth Very sensitive to motion Limited field of view Standardization challenge Time-consuming data analysis Dye needed for IVM High cost

OSP and SDF provide morphologic (diameter, density) and functional (true blood flow velocity in mm/s) evaluation of capillaries. Only superficial circulation is measured due to limited depth of penetration. Limited field of view can be another disadvantage, making large area evaluation time-comsuming, if not impossible. DRSI and NIRS can offer oxygen and hemoglobin concentration of superficial soft tissues. Lower sensitivity and requirement of complicated computing methods are major disadvantages (88).

Ultrasound visualizes and estimate blood flow with anatomical reference. In contrast to the other optical methods, this method provides cross-sectional imaging with acceptable depth of penetration for oral mucosa. US is sensitive to the motion of RBCs and obtains velocity information that is a projection of the true velocity vector onto the acoustic beam. For over 15 years US emerges as a reliable and valuable tool for longitudinal assessment of soft and hard tissue healing and has proven to aid clinicians in decision-making at an individual level (19, 20, 100, 102). As with the other methods, there are limitations, such as provision of surrogate values for velocity and volume, reliance on coupling medium, and lower spatial resolution than optical images in general (Tables 5, 7, Figure 3). Previous limitations, may have been lifted with the introduction of a small form factor (toothbrush size) and high-frequency (25 MHz) ultrasound transducer suitable for practical intra-oral scanning (21–23, 34, 105). The costs of these technologies can be categorized as low, moderate, or high. Low-cost techniques are generally priced up to $20,000, moderate-cost technologies range up to $60,000, and high-cost systems are priced at $60,000 and above. High-cost methods, such as LSCI, and Spectral Imaging Methods are expensive due to the complexity of the technology and equipment. In contrast, moderate-cost options like LDF and Ultrasound systems are more affordable, with costs varying based on their features and specific applications. Low-cost techniques, such as Videocapillaroscopy and Videomicroscopy, are typically more budget-friendly but may have limitations in resolution and functionality compared to the more advanced methods. The total cost of each technique includes training, maintenance, and consumables. The cost-benefit ratio depends on the clinical or research needs, frequency of use, and required image resolution (Table 1).

### 4.5 Future directions

Imaging techniques for assessing wound healing have evolved significantly in recent years and have become important tools in understanding complex oral disease progression and healing processes. Early diagnosis of infection and inflammation as well as quantification of soft tissue healing could significantly improve future patient care and surgical outcome. Longitudinal assessment of wound healing in a range of surgical techniques, compromised patients, graft maturity, and complication detection may soon aid clinicians in establishing evidence-based (treatment) procedures. Bias and limitations of each technique evaluated, are inherently connected. For example, having 3D images instead of 2D images in US would make the field of view more robust. For LCSI, increasing the penetration depth, would allow for better comparison with ultrasound. For LDF, standardization of the applied pressure (from the probe) to the tissue could improve the repeatability of the image. Additionally, factors such as patient movement, the overall depth of the field, and variability in probe positioning would benefit from reduced sensitivity in relation to the estimated gingival blood flow, leading to more consistent and reliable results.

### 4.6 Limitations of the study

The limitations of this study include a variety of parameters used for estimating circulation, as well as the heterogeneity of the techniques employed. Additionally, the assessment of blood flow was conducted across different clinical scenarios, including both non-surgical/surgical, pre-clinical/clinical settings, and at varying time points, and clinical and preclinical. These factors introduce variability and impact the consistency and comparability of the results. As such, further research is essential to establish more consistent methodologies for more robust comparisons between the different techniques used for measuring gingival blood flow.

## 5 Conclusions

This systematic review highlights currently available *in vivo* imaging techniques for non-invasive quantification of periodontal blood flow for the monitoring oral of wound healing healing and classifying diseases. Understanding the mechanisms, readouts, advantages, and limitations of each technique allows for the selection of an appropriate imaging method. Even though having been used extensively in medicine, these techniques are only used for research without much penetration into clinical care. Future investigations should focus on the longitudinal assessment of tissue perfusion after surgical procedures of differing incisions and flap designs to understand the implications of their use. Mapping and quantifying microcirculatory gingival pattern variations and their modifying factors could provide evidence-based treatment planning procedures. Using these tools could result in more predictable surgical outcomes and therefore enhance tissue healing and patient-centered outcomes. Cross-sectional US offers sufficient depth of penetration for oral soft tissue, can complement other optical methods that primarily evaluate superficial perfusion.

## Supplementary material

Supplementary Table S1 Quality assessment: Assessment for non-RCT (ROBINS-I).

Supplementary Table S2 Quality assessment: Assessment for RCT (ROB2).
