# Supplementary material for: Non-invasive and quantitative methods for assessment of blood flow in periodontal and oral soft tissues: a systematic review
Source: Front Dent Med. 2025 May 22;6:1587821. doi: 10.3389/fdmed.2025.1587821 (PMC12137316; doi:10.3389/fdmed.2025.1587821)
Supplement: Supplementary Table S1 — Quality assessment: Assessment for non-RCT (ROBINS-I). [file Table1.docx]

**Supplemental Table 1.** Quality assessment: Assessment for non-RCT (ROBINS-I)

| **Bias Assessment** | **Confounding bias** | **Selection of participants** | **Classification of interventions** | **Deviations from intended interventions** | **Missing data** | **Measurement of outcomes** | **Selection of the reported result** | **Overall bias** |
| --- | --- | --- | --- | --- | --- | --- | --- | --- |
| **Study** |  | **Risk of bias judgment (Low/moderate/serious/critical/NI)** | | | | | | |
| Heitzer et al. 2025 | Y | Y | Y | PN | PN | PN | PN | Moderate |
| Katz et al. 2024 | PY | Y | Y | PN | PY | PY | PN | Low |
| Diehl et al. 2022 | PY | PN | Y | PN | PY | PN | PN | Low |
| Liu et al.  2022 | PY | PY | Y | PN | N | PY | PN | Critical |
| Miron et al. 2022 | PY | PY | N | PN | PY | N | PN | Moderate |
| Svetlana et al. 2022 | PY | PN | Y | PN | PY | N | PN | Low |
| Komaki et al. 2022 | PY | PY | Y | PY | N | N | PN | Critical |
| Laredo-Naranjo et al. 2021 | PN | PY | Y | PN | Y | N | PN | Low |
| Yamamoto et al. 2021 | PY | PY | Y | PN | N | N | PN | Serious |
| Kuraji et al. 2019 | Y | PY | Y | PN | Y | PN | PY | Moderate |
| Alssum et al. 2017 | PY | PN | Y | PN | Y | N | PN | Low |
| Kaner et al. 2017 | PY | PN | Y | N | N | N | PN | Low |
| Ogino et al. 2017 | Y | PY | Y | PN | N | Y | PN | Moderate |
| Tatarakis et al. 2017 | PN | PY | Y | N | PY | N | PN | Low |
| Le Bars et al. 2016 | PN | PY | Y | PN | Y | PN | PY | Moderate |
| Reuther et al. 2016 | Y | PY | Y | PN | N | Y | PY | Serious |
| Svalestad et al. 2014 | Y | PN | Y | N | Y | N | PN | Low |
| Kawaai et al. 2013 | Y | PY | Y | N | Y | Y | PY | Critical |
| Kozlov and Ibragim 2011 | Y | PY | Y | PN | PN | PN | PY | Serious |
| Okada et al. 2010 | PY | PN | Y | N | Y | N | PN | Low |
| Sakr et al. 2010 | Y | PY | Y | PN | Y | PN | PY | Low |
| Svalestad et al. 2010 | Y | PY | N | N | N | N | PY | Serious |
| Singh et al. 2008 | Y | PY | Y | N | PY | PN | PY | Moderate |
| Retzpei et al. 2007a | PY | PN | Y | N | Y | N | PN | Low |
| Retzepi et . 2007b | Y | PN | y | PN | PY | PN | PN | Low |
| Rodriguez-Martinez et al. 2006 | Y | PY | Y | N | Y | N | PN | Low |
| Donos et al. 2005 | PY | PY | Y | PN | PY | PY | PY | Serious |
| Kocabalkan and Turgut 2005 | Y | PY | Y | N | Y | N | PY | Moderate |
| Patino-Marin et al. 2004 | Y | PN | PN | PN | Y | N | N | Low |
| Kerdvongbundit et al. 2003 | Y | PN | Y | N | Y | N | PN | Low |
| Kemppainen et al. 2003 | Y | Y | PN | Y | N | PN | Y | Serious |
| Akazawa and Sakurai 2002 | Y | PY | PN | Y | Y | N | PY | Serious |
| Ambrosini et al. 2002 | PY | PN | Y | N | PY | N | N | Low |
| Vag and Fazekas 2002 | PY | Y | Y | Y | PN | Y | Y | Critical |
| Kerdvongbundit and Vongsavan et al. 2002a | PN | PY | Y | PY | PY | PN | Y | Moderate |
| Kerdvongbundit and Vongsavan et al. 2002b | Y | PN | Y | PN | PY | N | N | Low |
| Heckmann et al. 2001 | Y | Y | Y | N | PY | N | PN | Low |
| Matsuki et al. 2001 | Y | PY | Y | PY | PY | PN | N | Moderate |
| Heckmann et al. 2000 | Y | PY | Y | PN | PY | N | N | Low |
| Ahn and Pogrel 1998 | Y | Y | Y | Y | PY | PY | PY | Serious |
| Perry et al. 1997 | Y | PN | Y | PN | PY | N | N | Low |
| Ketabi and Hirsch 1997 | PN | PN | Y | PY | PY | N | PN | Low |
| Schmid-Schönbein et al. 1997 | PY | PY | Y | PN | Y | N | PY | Moderate |
| Herlofson et al. 1996 | PY | PY | Y | PN | PY | PN | PN | Low |
| Hinrichs et al. 1995 | Y | Y | Y | PN | Y | N | PN | Low |
| Dodson et al. 1994 | Y | PY | Y | PN | Y | PN | PY | Moderate |
| Hoke et al. 1994 | Y | Y | N | Y | PY | PN | Y | Serious |
| Matheny and Johnson et al. 1993 | PY | Y | Y | PN | PY | PY | PN | Moderate |
| Baab et al. 1990 | PY | PN | Y | PN | Y | PN | PN | Low |
| Baab and Öberg 1987 | Y | Y | Y | PN | PY | PN | PY | Moderate |
| Baab et al. 1986 | Y | Y | Y | PY | PY | PN | PY | Moderate |
| Vág and Mikecs. 2022 | PY | PN | Y | PY | PY | PN | PY | Moderate |
| Mikecs et al. 2021 | Y | Y | Y | N | PY | PN | Y | Serious |
| Amaral et al. 2020 | PY | P | PY | PN | PY | PN | PY | Moderate |
| Fazekas et al. 2019a | PN | PN | Y | PN | PY | PN | PN | Low |
| Fazekas et al. 2019b | PY | PN | Y | PN | PY | PN | PN | Low |
| Molnar et al. 2019 | PY | Y | Y | N | PY | N | PY | Moderate |
| Fazekas et al. 2018a | PY | PN | Y | PN | PY | PN | PY | Low |
| Fazekas et al. 2018b | PY | Y | Y | PY | PY | PN | PY | Serious |
| Gànti et al. 2018 | PY | PN | Y | N | PY | PN | PN | Low |
| Molnar et al. 2018 | PY | PN | Y | PN | PY | PN | PY | Low |
| Molnar et al.  2017 | Y | PN | Y | PN | PY | PN | PY | Low |
| Tang et al. 2025 | PY | PN | N | PN | PY | PY | PN | Critical |
| Ooms et al 2023 | PY | PN | Y | PN | Y | N | PN | Low |
| Preidl 2021 | PN | PN | Y | PN | Y | PN | PY | Low |
| Sekhar Prasanth et al. 2013 | PY | Y | Y | Y | PN | PN | PY | Serious |
| Bodo et al. 2013 | Y | Y | Y | PY | PY | N | PY | Critical |
| Milstein et al. 2009 | Y | PY | Y | PY | PN | PN | PY | Critical |
| Zakian et al. 2008 | PY | PN | Y | Y | PY | PN | PN | Low |
| Lindeboom et al. 2006 | PN | PN | Y | PY | PY | PN | PN | Critical |
| Samal et al. 2024 | PY | PY | PY | PN | PY | PN | PN | Moderate |
| Sirinirund et al. 2023 | Y | Y | Y | PN | PY | PN | PY | Low |
| Barootchi et al. 2022 | PY | Y | Y | PN | Y | PN | PN | Low |
| Siqueira et al. 2021 | PY | PN | Y | PN | Y | PN | PN | Low |
| Tavelli et al. 2021 | Y | PN | Y | PN | PY | N | PN | Moderate |
| Chan and Kripfgans 2020 | PY | PY | Y | PY | PY | PN | PN | Critical |
| Izetti et al. 2020 | PY | PY | PY | PN | PY | PY | PN | Critical |
| Tikku ete al. 2010 | PY | Y | Y | PN | PY | N | Y | Low |
| Takeda et al. 2023 | PY | PN | Y | PN | Y | N | PN | Moderate |
| Townsend 2022 | PY | Y | Y | PN | Y | PN | PY | Critical |
| Yilmaz and Atlas 2021 | PY | PY | Y | PN | PY | N | PY | Moderate |
| Le et al. 2018 | Y | Y | Y | PY | PN | PN | PY | Moderate |
| Bastos et al. 2016 | Y | Y | PN | PY | PY | PY | PY | Critical |
| Janovszky et al. 2014 | Y | Y | Y | PY | PY | PN | PY | Low |
| Scardina et al. 2014 | PY | PN | Y | PN | Y | N | PN | Low |
| Takanos et al. 2010 | Y | Y | Y | N | PN | PN | PN | Moderate |

*Abbreviations: Y; Yes, PY; Probably Yes, N; No, PN; Probably No, NI; No Information.
