# Supplementary material for: Non-invasive and quantitative methods for assessment of blood flow in periodontal and oral soft tissues: a systematic review
Source: Front Dent Med. 2025 May 22;6:1587821. doi: 10.3389/fdmed.2025.1587821 (PMC12137316; doi:10.3389/fdmed.2025.1587821)
Supplement: Supplementary Table S2 — Quality assessment: Assessment for RCT (ROB2). [file Table2.docx]

**Supplemental Table 2. Quality assessment: Assessment for RCT (ROB2)**

| **Bias Assessment** | **Randomization** | **Identification and Recruitment** | **Deviations from intended Interventions** | **Missing data** | **Measurement of the outcome** | **Selection of the reported result** | **Overall bias** |
| --- | --- | --- | --- | --- | --- | --- | --- |
| **Study** | **Risk of bias judgment (Low/some concerns/moderate/serious/critical/NI)** | | | | | | |
| Tavelli et al. 2025 | Y | N | Y | Y | N | Y | Low |
| Cusack et al. 2025 | Y | N | Y | Y | N | Y | Low |
| Palombo et al. 2024 | Y | N | PY | Y | N | Y | Low |

*Abbreviations: Y; Yes, PY; Probably Yes, N; No, PN; Probably No, NI; No Information.
